# Towards Consistency in Geometry Restraints for Carbohydrates in the Pyranose form: Modern Dictionary Generators Reviewed

**DOI:** 10.2174/0929867328666210902140754

**Published:** 2022-01-01

**Authors:** Robbie P. Joosten, Robert A. Nicholls, Jon Agirre

**Affiliations:** 1 Oncode Institute and Division of Biochemistry, Netherlands Cancer Institute, Plesmanlaan 121, 1066 CX Amsterdam, The Netherlands; 2 Structural Studies, MRC Laboratory of Molecular Biology, Francis Crick Avenue, Cambridge CB2 0QH, England; 3 York Structural Biology Laboratory, Department of Chemistry, University of York, YO10 5DD, England

**Keywords:** Geometry restraints, ring conformation, pyranose, structural biology, dictionaries, refinement

## Abstract

Macromolecular restrained refinement is nowadays the most used method for improving the agreement between an atomic structural model and experimental data. Restraint dictionaries, a key tool behind the success of the method, allow fine-tuning geometric properties such as distances and angles between atoms beyond simplistic expectations. Dictionary generators can provide restraint target estimates derived from different sources, from fully theoretical to experimental and any combination in between. Carbohydrates are stereochemically complex biomolecules and, in their pyranose form, have clear conformational preferences. As such, they pose unique problems to dictionary generators and in the course of this study, require special attention from software developers. Functional differences between restraint generators will be discussed, as well as the process of achieving consistent results with different software designs. The study will conclude a set of practical considerations, as well as recommendations for the generation of new restraint dictionaries, using the improved software alternatives discussed.

## 1 Introduction

The two most prolific macromolecular structure determination techniques, X-ray crystallography and electron cryomicroscopy, produce three-dimensional maps that, when data are of sufficiently high quality, allow the almost unequivocal positioning of atoms in a molecular model. Factors such as excessive thermal movement, crystal disorder, sample heterogeneity, or even the instability of the incident particle beam used – be it composed of photons or electrons – can all have a detrimental effect on data quality. With decreasing resolution, prior knowledge informs macromolecular refinement with increasing relevance – controlled by weighting parameters that are specified by the user or determined algorithmically. Prior knowledge is introduced in the form of geometric restraints which, depending on the overall weight – applied to the experimental term in a typical refinement target (see Equation 1) – can have anything from a mild stabilising effect on the refinement of model parameters to controlling the fine-tuning of the atomic positions, interatomic bond distances, or even the final tentative shape of a refined molecule. Indeed, from the ring conformation of a monosaccharide to the secondary and tertiary structure of a protein domain, anything can be directed towards a pre-established template; a notion of correctness enforced by the affirmative accumulation of coherent views of a molecule in its resting state, should there be such a thing in a biological system.

(Eq. 1) L(p)=wLX(p)+LG(p)

Equation 1: L(p) is the overall target to be optimised, which includes a target function representing the agreement between model and experimental data, LX(p) – whose predominance can be controlled by a weighting term (w) – and a separate component LG(p) that penalises deviations from ideal geometry.

### 1.1 How Chemistry Rescued Structural Biology

On the provenance of prior knowledge, different sources may be used. Whereas coarse-grained or protein-specific information – secondary, tertiary, or quaternary structure in proteins, conformational dependence of the protein backbone geometry, to name a few – can only be mined from macromolecular structure databases, it has been proven time and again that finer chemical details that are not dependent on macromolecular context can be inferred from small molecule structure databases, which have much higher coordinate precision than their macromolecular counterparts. From the original 1982 survey of the Cambridge Crystallographic Database by Taylor and Kennard [1], going through the 1991 Engh and Huber [2] study of the Cambridge Structural Database [3] (CSD), to the latest open-source efforts of Long and collaborators [4] mining the Crystallography Open Database [5] (COD), successive refinements of the geometric descriptions of monomers have provided better predictions of what the bond lengths, angles, and sometimes torsions may be in macromolecular structures. Crystallography has over the years provided a useful test for restraint sets via the free-R set of reflections: holdout-validation, used as a means of evaluating the predictive power of a structure model and thus also the underlying restraints. While the differences between methods’ estimates of a single C-O bond length may seem negligible – typically of the order of 1/100th of an Ångström – the cumulative effect that they have can improve R-factors, model phases, and the resulting electron density maps. In the particular case of saccharides, which are the main concern of this work, their contribution may range from moderate to substantial. While ligands and polysaccharides will not typically contribute many atoms to the total, oligosaccharides in heavily glycosylated proteins may well account for 10% of the atoms in a model [6]. Therefore, there is a strong case for obtaining the best possible geometric estimates, particularly considering how subpar restraints have affected aspects of carbohydrate model geometry such as ring conformation and fit to electron density in the past [7–9]. Indeed, model building programs such as *Coot* [10] have recently introduced modernised dictionaries in an attempt to mitigate these issues.

Despite their widespread use by dictionary generation programs, small molecule databases are not the only source of chemical information that can be used to gather estimations. Modern software (*vide infra*) may run a short molecular mechanical or quantum mechanical energy minimisation, deriving their estimations from the distribution of bond lengths and angles that are visited during the minimisation, along with the estimated standard deviations (e.s.d.’s) that are reported in restraint dictionaries. Even though these methods might improve in the future, previously, they have been found to disagree with what seems to be a near-consensus between those dictionary generation methods relying on small molecule databases [11, 12]. However, as the next section will reveal, force fields and molecular mechanics are not only relevant to restraint generation; indeed, they are most useful for the generation of conformers.

### 1.2 Restraint Dictionaries Contain more than just Restraints

**Fig. (1):**
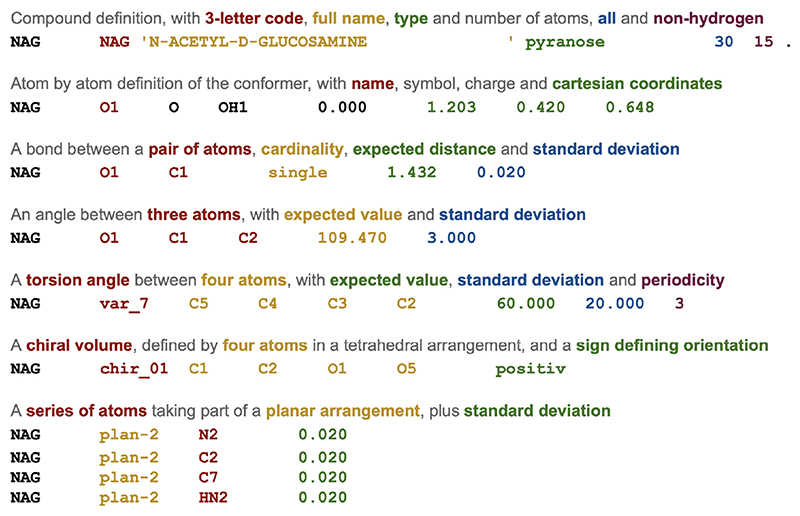
Organisation of chemical knowledge in a CIF restraint dictionary. This is an extract of the current CCP4 dictionary for N-acetyl β-D-glucosamine, GlcNAc, which is represented in the PDB database as ‘NAG’. The first row (compound definition) is unique per dictionary file. The starting coordinates, which should represent the minimal-energy conformer, are stored in the atom by atom definition. Torsions, chiral volumes and planes are optional, and indeed many programs do not generate them. *(A higher resolution/colour version of this figure is available in the electronic copy of the article).*

As previously mentioned, dictionaries contain information about bond lengths between two atoms, angles between three atoms, and dihedral angles (torsions) between four atoms. A strictly complete set of these restraints, together with unique atom names, would be enough to recreate a molecule in 3D space and keep its chemical properties. However, this is an ideal which does not meet reality. In practice, restraint sets need not be complete, and individual restraints may be imprecise, inaccurate, wrong or inconsistent with the rest of the restraints. Therefore, other information is typically included: starting coordinates, definitions of planar groups, and chiral volumes; Fig. (1) shows a description of the different fields that can appear in a dictionary file in CIF (Crystallographic Information File) format, and the data types that are contained in them. While the former may be there for the convenience of model building programs – thereby being able to simply apply a rotation and translation to available coordinates – the other two provide information that should otherwise be part of a complete set of torsion restraints: *i.e.,* four atoms in a planar arrangement may be represented as either 180° (trans) or 0° (cis) dihedral angles, and chirality should follow the value and sign of the (improper) dihedral angles.

Torsion restraints may be harmonic – *e.g.*, having 2 or 3 targets – or unimodal. However, regardless of nature, they need to be assigned a starting value fitting one of the targets. These are typically computed from the starting coordinates so that the restraints will initially work towards keeping the initial conformation – presumed to be low-energy, most probable, and biologically relevant – and then simulate resistance as rotation around a bond leaves substituents in eclipsed conformations. Therefore, the correctness and eventual success of harmonic torsion restraints can be directly related to the starting coordinates. More concretely, to how closely the initial coordinates resemble the most probable state of the restrained small molecule. In the case of monosaccharides in pyranose form, which most often can be found in a chair conformation, generating a plausible initial set of atomic positions may not be as trivial as with other molecules. Depending on how the geometric optimisation of the conformer is done upon dictionary generation, improbable conformations such as envelopes or half-chairs may be generated. This has been reported to have happened in both restraint dictionaries – *e.g.*, those from the CCP4 monomer library [13, 14] – and even compound description dictionaries such as the ones found in the worldwide Protein Data Bank (wwPDB)’s Chemical Component Dictionary (CCD) [11]. Compound description dictionaries do not contain restraints and as such, place more emphasis on chemistry and initial coordinates than, *e.g.*, the CCP4 monomer library, which is focused on providing restraints. As a temporary fix, the *Privateer* carbohydrate validation software [15] has included additional functionality to check, patch, and extend torsion restraints in dictionaries – including unimodal torsion restraints for ring bonds. This allows users to restrain the correct pyranose conformations during the refinement of low-resolution structures of large glycoproteins [6, 16] and protein-carbohydrate complexes [17–19].

### 1.3 The wwPDB’s Remediation of Carbohydrates

On the subject of compound description dictionaries, which the wwPDB store and curate as part of the CCD (available online [20]), an interesting development concerning carbohydrates has taken place recently: all definitions of polysaccharides have been obsoleted, being replaced by their linked monosaccharide components. Also, monosaccharide components have seen their atom names standardised following IUPAC style residue naming and standard atom nomenclature following IUPAC-IUBMB recommendations. For example, β -D-xylopyranose (three-letter code ‘XYP’ in the CCD) has seen its atom names changed from ‘C1B’ (anomeric carbon) and ‘O5B’ (in-ring oxygen) to ‘C1’ and ‘O5’ respectively. The wwPDB CCD is considered the standard reference for chemical components in structural biology, and, as such, existing dictionaries were/are being adapted to match the remediated atom definitions.

Another consideration that pertains to the wwPDB’s handling of carbohydrate structures is that models need to be validated in order to eliminate gross mistakes upon PDB deposition and give the community an idea of structural quality [21]. Currently, wwPDB validation uses CCDC Mogul [22], allowing finely tuned searches of a particular bond’s average length and standard deviations – likewise for bond angles and torsions – calculated from the CSD’s collection of small molecule structures. Indeed, considering the comparably low resolution of macromolecular crystallographic structures, most of the deviations from ideal geometry found in carbohydrate moieties will be a product of uncertainty. While geometric distortions may represent a real feature of macromolecular models, they are the product of a rare event where a strained conformation occurs in an otherwise stable crystalline structure; hence, outliers are rarely informative and largely misleading.

The quest for realistic chemical geometry has diversified in the last decade with the advent of faster molecular simulation methods and the use of accelerated graphics processing architectures for general purpose calculations. Simulation procedures can now inform traditional restraint generation or completely substitute it in either an interactive [23] or non-interactive [24, 25] way. Yet simulations are not the only way around traditional procedures: external restraints can supplement or override finely grained geometric information with coarse-grained distances based on comparative analyses [26, 27]. Still, restrained refinement is commonplace and typically the first option to try right after model building, making dependable geometric restraints a key tool in the process.

While restraint-producing programs have been exhaustively compared in the past [12], currently, there is no study of the application of contemporary methods to the generation of restraints for carbohydrates in their pyranose form. As saturated rings, a number of additional complexities need to be dealt with: ring conformation, differences in bond lengths and angles due to atoms being in a ring shape, and what torsion restraints are put in place. Furthermore, due to the wwPDB’s deprecation of polysaccharide codes, new functionality is urgently needed to restrain glycosidic linkages that were previously part of polymer dictionaries.

In the present work, we analysed the strengths and limitations of several state-of-the-art restraint generation programs in regard to pyranose carbohydrates, of which a set of representative molecules were chosen and tested. All the programs discussed here have modern, maintainable architecture, and are under active development, meaning that swift improvements can be made. Following an initial analysis with unexpected results, we reached out to the developers of the software we tested, and, where needed, software updates were made, which we then re-evaluated. The changes made and the degree of consistency – as measured by the absence of wildly differing results in the produced restraints and optimised conformers – achieved in the final results have greatly improved the way these programs handle carbohydrates in pyranose form and serve to highlight the impact that feedback can have on the development of scientific software.

## 2 Methods

### 2.1 Software Chosen for this Study

Restraint dictionaries and initial conformers were generated using different restraint generators: based on the limited diversity of results from a broader comparison study [12], a choice was made to pick Grade [28] from Global Phasing [29] (webserver version at the time of submission: Release v1.106 Dec 11, 2019), eLBOW [30] from the Phenix suite [31] (v1.19.2-4158), Coot’s Pyrogen (0.0-pre-rev10459) and AceDRG [32] (v226) from the CCP4 suite [14] for this study. The generation procedure used is documented hereafter. A decision was made not to include Libcheck [13] or PRODRG [33] based on the fact that they have not been actively developed recently. Like Pyrogen, eLBOW is able to connect to a local installation of CSD Mogul [34] – for this study; version 2020.3.0 was used. Unlike the others, eLBOW allows for the use of different backends or engines for the generation of restraints and coordinates, including the ability to plug in a number of third-party packages for quantum chemistry calculations. In addition to running Grade, eLBOW, Pyrogen, and AceDRG using default settings, we also included two additional modes of eLBOW, which involve the use of a small molecule database (Mogul), and a semi-empirical QM method (eLBOW’s native RM1/AM1 [35, 36] implementation).

Consequently, we consider a variety of approaches that may be categorised as QM-based (eLBOW; eL-BOW-RM1/AM1) or small molecule-based (AceDRG; eLBOW-Mogul; Grade; Pyrogen), the latter of which can be further sub-categorised as being derived from the COD (AceDRG) or the CSD *via* Mogul (eLBOW-Mogul; Grade; Pyrogen). In addition to the underlying data source, another relevant difference between small molecule-based tools, in the context of restraint generation, lies in the selection and filtering of representative observations in the small molecule data. The four tools use different implementations for geometric optimisation of the final conformer.

### 2.2 Test Cases

The dictionary generators tested here consist of algorithms and underlying data; The algorithms interrogate the underlying data in such a way that only finite and deterministic answers can be retrieved – *i.e.*, for one particular software, the distance between two linked carbon atoms in a pyranose ring will be exactly the same across all epimers and most derived monosaccharides. Therefore, to avoid ending with a collection of thousands of identical data points, a decision was made to choose representatives that would test the capabilities of the algorithms.

**Fig. (2):**
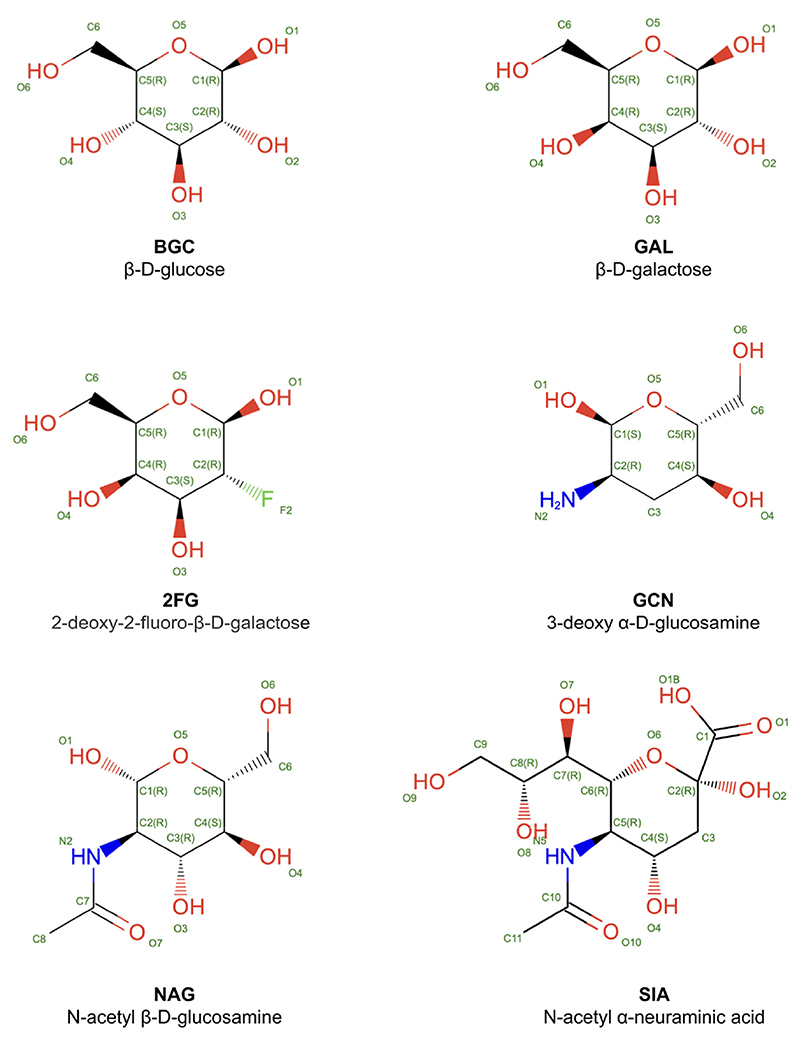
2D diagrams of the carbohydrates used as test compounds, together with their PDB Chemical Component Dictionary three-letter codes and chemical names. The atom names used in PDB/mmCIF files are annotated in green. The individual panel images have been obtained and used under permission from RCSB PDB [55, 56]. *(A higher resolution/colour version of this figure is available in the electronic copy of the article).*

A number of monosaccharides were chosen with increasing chemical complexity (see Fig. 2): β -D-glucopyranose (code ‘BGC’ in the wwPDB’s CCD), the simplest case for conformer generation due to having all substituents in equatorial position; β -D-galactopyranose (‘GAL’) as a C4 epimer of glucose; 2-deoxy-2-fluoro β-D-galactopyranose (‘2FG’) as a variation incorporating fluorine (chosen because it contained a substitution with an atom other than the usual C, N, O, and H, which might pose complications to software); 3-deoxy α-D-glucosamine (‘GCN’), which lacks a -OH at position 3 and incorporates an amine group, N-acetyl β-D-glucosamine (‘NAG’), the most frequently modelled monosaccharide in the PDB and which naturally expands on the previous one; and N-acetyl α - neuraminic acid (‘SIA’), as a complex L-ketopyranose with acidic character and great biological importance [37, 38].

### 2.3 Dictionary Generation Procedures

All the selected programs incorporate a function for generating restraints for a wwPDB CCD entry, using the mmCIF file that contains a chemical description of the molecule. In order to make the results as comparable as possible, this option was selected as the input chemical descriptor.

#### 2.3.1 AceDRG

AceDRG derives stereochemical information from the COD using an atom typing system that encapsulates the local structural environment. This is conceptually similar yet technically distinct from that used by Mogul when extracting information from the CSD. AceDRG uses RDKit [39] for chemistry perception and conformer generation and uses REFMAC5 for final geometry optimisation.

#### 2.3.2 eLBOW

eLBOW’s default mode derives restraints using a simple force field derived from quantum chemical calculations performed on pairs of main-group atoms. Conformer geometry optimisation is performed using CCTBX [40]. Refer to the eLBOW reference for further details [30].

#### 2.3.3 eLBOW-Mogul

Running eLBOW in Mogul mode (keyword: “--mogul”) results in a dramatically different approach being used compared with the other (QM-based) modes available in eLBOW. Interestingly, this allows for more direct comparison with the other tools that also derive restraints from small molecule data. This mode requires a connection to a local installation of the CCDC program suite, which includes Mogul.

#### 2.3.4 eLBOW-RM1/AM1

In addition to supporting several third-party quantum chemistry packages, eLBOW includes an implementation that uses the RM1/AM1 semi-empirical quantum chemical method (keyword: “--opt”). We included this mode due it being recommended over the default behaviour.

#### 2.3.5 Grade Web Server

Grade connects to the CSD through Mogul to get reference data, but in cases where insufficient observations are available, this is supplemented by semi-empirical QM calculations using RM1. Grade is also available through a web server which allows users without the CCDC suite to generate restraints. All compounds were processed on the Grade web server using the “Produce dictionary for an existing PDB chemical component” option. All other options were kept at the server’s defaults.

#### 2.3.6 Pyrogen

The *Coot* [41] model building software package is ubiquitous and nearly fully featured. As such, it incorporates various tools to aid the building and validation of ligands, including a 2D builder and diagram generator (Lidia) and a restraint generator (Pyrogen) [42, 43]. Like eLBOW-Mogul, in default mode, it requires a connection to a local installation of the CCDC program suite, which includes Mogul (noting that Pyrogen has an execution mode that does not require Mogul; Pyrogen can use MMFF as a fallback). Like AceDRG, conformer generation is performed using RDKit, although, in contrast, geometry optimisation is performed using Coot’s internal minimiser.

### 2.4 Analysis and Comparison of Geometry Restraints

#### 2.4.1 Bond Lengths and Tolerances

**Fig. (3):**
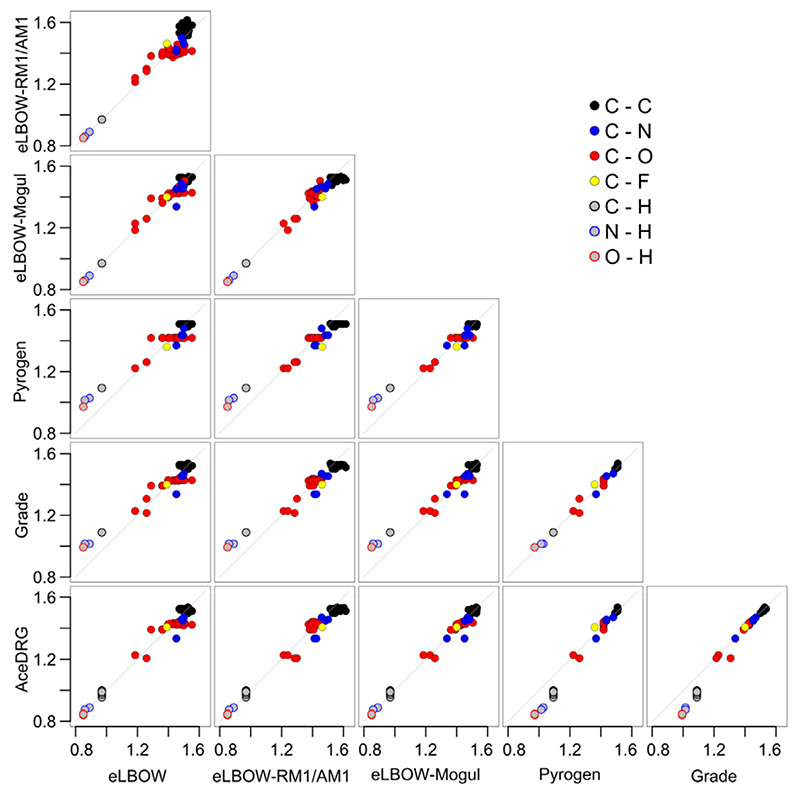
*Versus* plots for equivalent bond lengths across the different programs. Distances are specified in Ångströms. Black circles represent carbon-carbon bonds at the coordinates specified by the restrained bond lengths in the two compared programs; blue corresponds to carbon-nitrogen, red is carbon-oxygen, yellow is carbon-fluorine, and grey encompasses all bonds between hydrogen and any other atom. Plot style inspired by Figure 5 in Steiner & Tucker [12]. *(A higher resolution/colour version of this figure is available in the electronic copy of the article).*

**Fig. (4):**
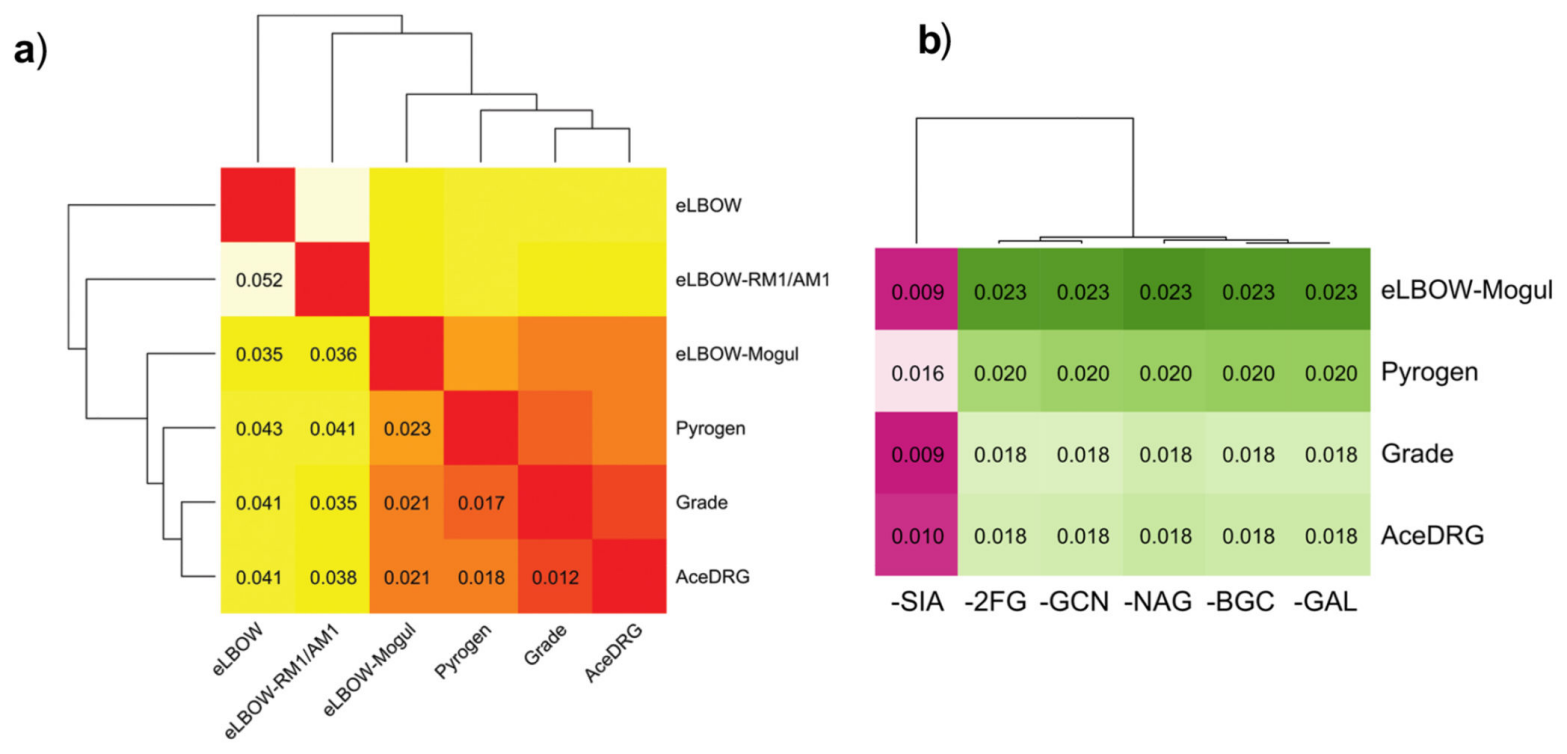
Comparison of bond length restraint target values from different dictionary generators, excluding bonds involving hydrogen atoms. Vertical and horizontal dendrograms illustrate the result of complete linkage hierarchical clustering of the rows and columns, respectively. **a**) All-on-all comparison of the six dictionary generators, showing RMSD values calculated over all corresponding bonds in all six components considered in the study. The red-orange-yellow-cream colour gradient indicates increasing RMSD values. **b**) Analysis of the influence of individual compounds on bond length RMSD values, for the four programs that use data derived from small molecule databases (eLBOW and eLBOW-RM1/AM1 were excluded, so as to focus on a detailed comparison of the four more similar programs). Values correspond to Jacknife estimates of the bond RMSD between each program versus the other three programs, after removal of the component with CCD ID listed on the horizontal axis. The purple-cream-green colour gradient indicates increasing values. *(A higher resolution/colour version of this figure is available in the electronic copy of the article).*

**Fig. (5):**
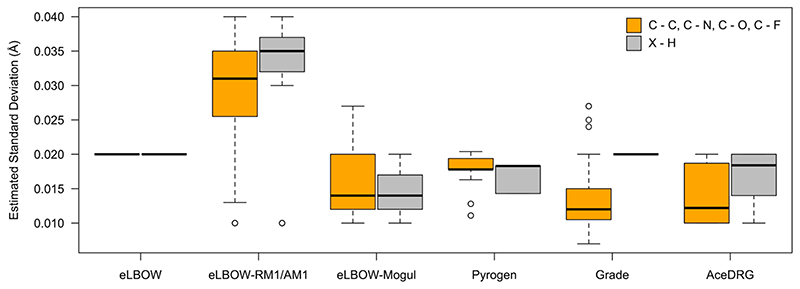
Boxplots showing the distributions of bond length e.s.d. values output by the six compared dictionary generators, for the six CCD components considered in the study. Separate boxplots are shown for bonds that do not involve a hydrogen (orange), and those between any atom and a hydrogen (grey). *(A higher resolution/colour version of this figure is available in the electronic copy of the article)*.

**Fig. (6):**
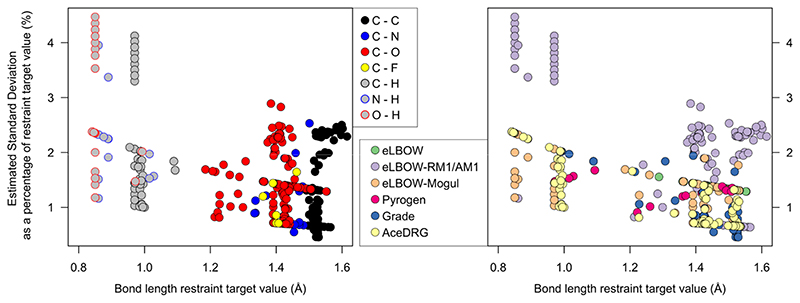
Relationship between bond length restraint target and e.s.d. values. Both plots show the same data, but coloured differently - by bond type (left; using the same colour scheme as in Fig. 3), and by dictionary generation program (right). *(A higher resolution/colour version of this figure is available in the electronic copy of the article).*

The generated restraint files were analysed, and the matching restraints were classified by type of bond and compared (Fig. 3). The RMSD between the different restraint sets was calculated and used to generate a heatmap and dendrogram, illustrating the overall comparative (dis)similarity of the restraint sets generated by the different programs (Fig. 4). Boxplots were created to show the distributions of bond length e.s.d. values for each generator, which are used for the relative weighting of individual restraints during refinement (Fig. 5). The relation between bond length and e.s.d. was investigated with respect to the bond type and the restraint generator (Fig. 6).

#### 2.4.2 A Note on Torsion Angles

Due to the disparity of behaviours exhibited by the different programs with regards to torsion restraints (unequal number of restraints or no restraints at all, and wildly differing initial angles), a decision was made not to compare them but rather regard these differences as design choices made by the developers of the programs. Users are advised that if they are considering using torsion restraints in refinement, they should check the restraint files for target values, as shown in Fig. (1). Alternatively, a new torsional set containing conformation-enforcing restraints may be generated using the *Privateer* software [15].

#### 2.4.3 Validation of the Conformer in the Starting Coordinates

The starting coordinates in dictionaries represent the atoms that are placed upon model building, and should ideally represent the most frequent conformation the monomer will be modelled in – a chair in the case of pyranose sugars. The *Privateer* software [15] was run on the PDB files produced by the dictionary generation software in order to extract the Cremer-Pople parameters [44] for the ring conformation of the conformers. These were then compared with those stored in *Privateer*’s database, which was originally computed from manually curated idealised coordinates from the CCD. In addition to ring conformation, *Privateer* also checks that the absolute stereochemistry and anomeric form detected from the coordinates match the CCD’s definition for the monosaccharide. It should be noted that a conformer showing the most favourable ring conformation needs not represent the exact minimum-energy conformation, as this will depend more finely on certain factors that remain unknown at the time of dictionary generation, such as the position of the hydrogen atoms, which are not observed in macromolecular crystallographic data, but deduced based on the carbohydrates environment, particularly with respect to potential hydrogen bonding partners.

## 3 Results And Discussion

The results presented here will aim to illustrate the differences and similarities between the produced dictionaries, focusing on the sections that are common to all programs, such as bond lengths, angles, planar groups and chiralities, and the set of coordinates. It should be noted that an end user is not expected to interact with dictionaries in this way; rather, users are encouraged to monitor disagreement between measured distances and angles and their ideal values in the produced dictionary. This can be done, for instance, interactively in Coot *via* the ‘Ligand’ menu.

### 3.1 Molecular Perception

#### 3.1.1 Protonation

Within the test compounds, SIA and GCN have functional groups that are subject to pH dependent (de)protonation. This has repercussions for restraint generation as bond orders, charges, and, naturally, the presence of hydrogen atoms in the model are dependent on the protonation state. According to the CCD entry of SIA, the carboxyl group is protonated despite a pKa of 2.6 [37]. As a result, the bonds for the carboxyl oxygens are marked as single and double. The perception of this acidic group differs amongst the restraint generators: Grade accepts the protonation state as-is and keeps the bond orders, giving target lengths of 1.307 Å and 1.215 Å, respectively. AceDRG retains the bond orders despite removing the hydrogen bond and setting both bond lengths to 1.207 Å. eLBOW and Pyrogen assign delocalised bond types and remove the hydrogen. Notably, the QM-based restraints from eLBOW have slightly different targets for the two bond lengths in the acidic group.

The amine on GCN is described as not protonated in the CCD. This is generally followed by the restraint generators, with the exception of Pyrogen, which protonates the amine.

When it comes to protonation, there seem to be two schools of thought: keep things as-is or predict the protonation state based on some algorithmic logic (which would presumably be pH-dependent). We observe that the latter approach can cause differences between programs, which would be an argument for the first approach. At the same time, this does require that the input data describes the most likely protonation state, which is clearly not the case for the CCD description of SIA. Perhaps the most robust approach would be to have a dictionary that describes the most likely protonation state but has modifiers that support the possible other protonation states. Indeed, the PDBx/mmCIF format includes chemical component modification categories that could facilitate such a mechanism, allowing the provision of restraints corresponding to multiple tautomeric states in a cohesive fashion. In doing so, this would force users (or the software they use) to make an informed decision about the protonation state to model. Furthermore, the adoption of such an approach would undoubtedly spur the development of improved tautomer building and validation tools.

#### 3.1.2 Bond Lengths and Tolerances

Comparing the different bond length targets per program shows that there are systematic deviations for bond lengths involving hydrogens (Fig. 3). Grade and Pyrogen produce consistently longer X-H bonds. This may be traced back to the choice of the positioning of hydrogens either at the nuclear/proton position or at the electron cloud position. Grade and Pyrogen use the nuclear position of hydrogen atoms by default, which is indeed further away from the bonded heavy atoms. This setting can easily be changed in Grade. It should be noted that while the length of bonds involving hydrogens is typically not refined in macromolecules, the position of hydrogens does affect the way anti-bumping or Van der Waals restraints work, which has a knock-on effect on torsion angles.

When only bonds between non-hydrogen atoms are considered, a distinct clustering in restraint generators appears. Fig. (4a) shows a heatmap of RMSD values calculated between equivalent bond length restraint targets between programs but excluding bonds involving hydrogens. When this is captured in a dendrogram, a clade appears consisting of all the programs that obtain restraint targets by data mining of small molecule databases. Somewhat surprising is that the smallest difference is found between Grade and AceDRG, which use the CSD and COD as data sources, respectively. This indicates that, with appropriate data mining approaches, the two different small molecular databases can be used as equivalent data sources, at the very least for carbohydrates in the pyranose form. Surprising, however, is that the overall differences are dominated by the restraints for SIA (Fig. 4b). When this compound is not taken into account, the RMSD values drop substantially, showing that the consistency between restraint generators is very compound-specific. In this case, exclusion of SIA reveals that the eLBOW-Mogul bond restraints for the other compounds are actually much more consistent with the AceDRG, Grade, and Pyrogen dictionaries than would be interpreted from (Fig. 4a). The consistency of the six dictionary generators for individual components can be further inspected in Supplementary Figs. (1-6).

Individual bond length restraints are weighted by their e.s.d., which makes the perceived e.s.d. values from the different restraint generators very relevant. The distributions for bond length e.s.d. values, separated by bond type and programs (Fig. 5), show several features. First of all, the e.s.d. value distributions differ greatly between programs. Secondly, it is clear that the values are not bond-specific in eLBOW (default mode). That is, all bond length restraints are weighted equally. This is also the case with Grade, but only for bonds involving a hydrogen atom. This will have a much smaller impact, as the bond lengths involving hydrogens are typically constrained in macromolecular refinement, removing the individual weighting of these bond lengths. A third feature is that all e.s.d. values from eLBOW-RM1/AM1 are relatively high compared to the values from other software. This will have an effect on model refinement with restraints from eL-BOW-RM1/AM1: the restraints for non-standard compounds will be effectively much looser than those for other structural components such as amino acids and nucleotides. This will make it harder to balance the overall weight between the experimental data and the prior knowledge (Equation 1).

The relation between bond length target and e.s.d. is investigated further at the level of bond type and restraint generator (Fig. 6). Bonds involving hydrogens form two clusters in terms of length, each having large spreads in terms of e.s.d. Carbon-oxygen bonds have a large spread in both dimensions, which is understandable due to the different types of functional groups in which they are involved. Carbon-carbon bonds cluster very well in terms of bond length. There is only one type of carbon-fluor bond in our test set, and the assigned bond length target and e.s.d. from different restraint generators vary substantially. This might be caused by different data mining queries in the small molecule databases and the limited number of returned values. A potentially useful feature for all restraint generators would be to capture the number of observations on which a target is based. This would, however, require an extension of the PDBx/mmCIF format.

Apart from showing that e.s.d. values are relatively high for eLBOW-RM1/AM1, Fig. (6) also demonstrates the upper and lower limits in e.s.d. values implemented in some software, notably AceDRG. This reduces the distinction between different types of bonds in terms of restraint weighting, albeit to a lesser extent than fixing to a constant value as with eLBOW.

It is clear that the treatment of e.s.d. values has been subject to very different design choices amongst the different restraint generation software. Minor fluctuations in e.s.d values will generally have little noticeable effect on the refined atomic positions and thus on subsequent biological interpretation of the macromolecular model. However, such differences in weighting will affect local model validation and may sometimes explain geometric deviations that are flagged as outliers by external tools such as the wwPDB validation server. It should be noted that the dictionary generators are designed and developed alongside the model refinement program from the same suite. We speculate that it would be reasonable to expect the e.s.d. values reported in the restraint dictionaries to be strategised in line with the specific implementation in the matching refinement software. Consequently, special care must be exercised when switching between different suites during the model building and refinement procedure, especially when transferring bespoke restraint dictionaries.

#### 3.1.3 Chirality and Planarity

Carbohydrates have many chiral centres that are biologically relevant, so an accurate description of the handedness of a chiral centre is required. All restraint generators created equivalent chiral centre descriptions.

NAG and SIA contain groups of atoms that are expected to be in a planar structure. In NAG, this plane consists of the atoms C2, N2, HN2, C7, O7, C8. Similar to in a peptide, the bond between N2 and C7 has a bond order higher than 1. As a result, the atoms in the plane can take on a *cis*- and a *trans*-conformation with a strong preference for the latter [45]. The possibility of having two conformations is described in several ways: AceDRG splits the plane into 2 sections and defines the N2-C7 bond as rotatable with target values at 0° and 180°. Grade also defines two planes but locks the torsion angle in a *trans*-conformation. All three eLBOW modes use a single plane and lock the atoms into a *trans*-conformation. Pyrogen also defines a single plane but does allow both *cis*- and *trans*-conformations. SIA has an equivalent planar group (atoms C5, N5, HN5, C10, O10, C11) that is treated the same as in NAG by all restraint generators. It should be noted that planarity restraints still allow non-planar conformations in the refinement of cases where the experimental data are particularly strong.

The carboxyl group of SIA (atoms C2, C1, O1A, O1B) is treated as a single plane, without rotatable bonds by all programs. Grade also has the HO1B atom, which is included in a separate yet overlapping plane consisting of atoms C2, C1 O1B, and HO1B. The C1-O1B bond is defined as rotatable, with preferred angles 0° and 180° but with weak torsion restraints targeting either value. However, the hydrogen atom will be kept in the plane by the planarity restraints.

### 3.2 Plausibility of the Starting Coordinates

**Fig. (7):**
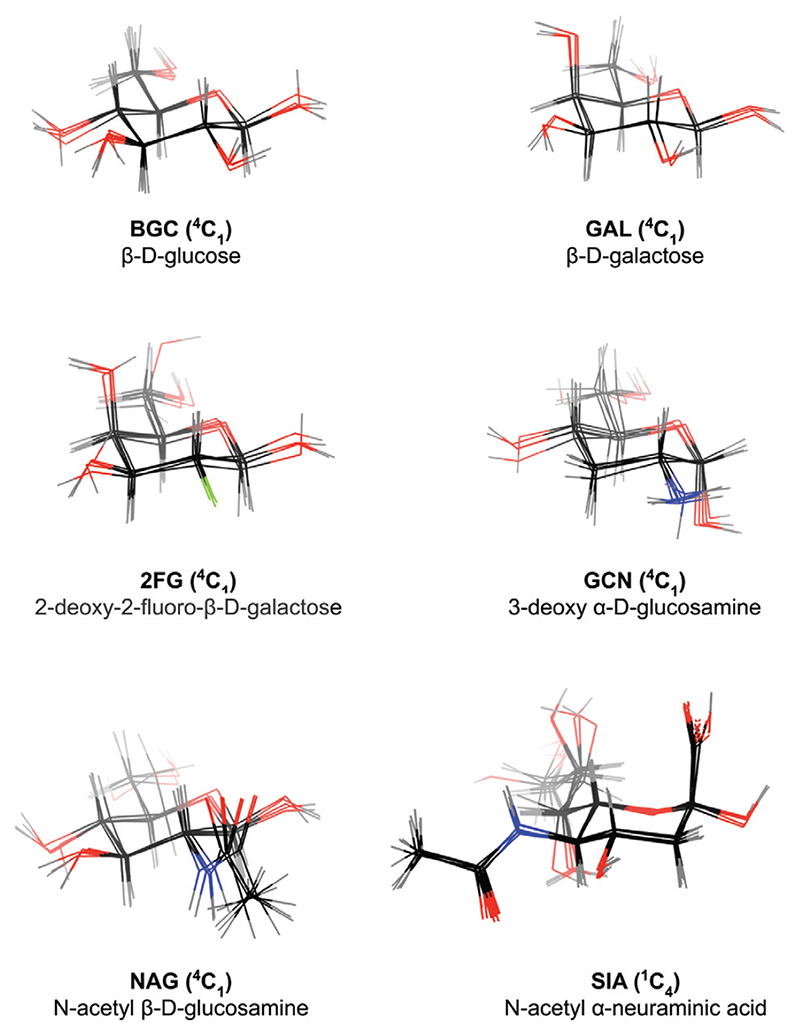
Superposition of the starting coordinates produced by all programs after they were improved by their authors. The expected minimal-energy ring conformations, shown as thick lines, represent the most probable conformations, thus making suitable starting coordinates for most cases. Figure and superposition produced with CCP4mg [46]. *(A higher resolution/colour version of this figure is available in the electronic copy of the article).*

*Privateer*’s validation of the produced conformers we produced initially is shown in Supplementary Table 1. All eLBOW protocols and Grade were able to obtain the expected minimal-energy ring conformation, a ^4^C_1_ chair for D-monosaccharides, and a ^1^C_4_ chair for sialic acid. Perhaps surprisingly, AceDRG produced skewboats for GAL, 2FG, NAG, and SIA. When operated with default parameters, Pyrogen’s conformer generation procedure did not arrive at energy-minimised conformations (5 out of 6 were high-energy conformers) and inverted the anomeric form of GCN and SIA. The superposed initial conformers are shown in Supplementary Fig. (7) (created with CCP4mg [46]), whereas the final conformers, obtained after the developers improved their conformer-generation algorithms following our feedback, are shown in Fig. (7) (also with CCP4mg [46]). Thick lines represent the consensusamong programs, reflecting the minimal-energy ring conformation, while thin lines show dissenting conformers. It should be noted that implemented improvements were general and not case-specific.

### 3.3 Disaccharides – Handling of Linkages

The proper description of covalent linkages between monosaccharides has drastically increased in importance due to the removal of polysaccharides from the CCD. Currently, most refinement programs use a predefined set of restraints for common glycosidic linkages (*e.g*., α(1-2) or β(1-4)), but less-common linkages require newly generated restraints. To our knowledge, AceDRG is currently the only discussed restraint generator that has dedicated procedures for creating linkage restraints [47]. We will therefore focus on more general issues with restraints of glycosidic linkages.

Glycosidic linkages are formed by condensation reactions in which a water molecule is created. That is, an oxygen atom of one of the monosaccharides, typically the O1 is removed. There were and still are many cases in which this oxygen removal was not properly modelled, resulting in a locally distorted model [48]. The most common issue with refining glycosidic linkages is related to the chirality around the anomeric centre. Since the PDB and mmCIF model file formats, in their current form, only describe the connectivity between two saccharides but not the intended chirality, users have to specify chirality indirectly. This can be done by using additional settings in refinement or by adding non-standard records to the PDB or mmCIF files. An additional option is that the chirality is inferred by the current model, but requires the model to be correct, which may be difficult with low-resolution data. A final option is inferring the chirality from the residue names of the saccharides, but this only works when these are correct. As all these options are error-prone, the wwPDB and the developers of restraint generation and refinement software are collaborating to create a robust solution, *i.e.,* capturing the full linkage description in the coordinate file.

In the meantime, we recommend that users working with polysaccharides specifically check glycosidic linkages in terms of connectivity, chirality, and torsion with tools such as *Privateer* [15], PDB-care [49], and CARP [50] and also through visual inspection using molecular graphics software.

## Conclusion

The conclusion and advice provided here relate to the present state of the software after they were updated as a consequence of our feedback. Our recommendations are general, as opposed to providing too many specifics regarding the execution of one tool or another; such details are better suited to software documentation or tutorials. We have covered the qualitative differences both in the output dictionaries and in the underlying methods behind various software tools. We do not claim superiority of one tool over another – rather, we aim to provide practical advice on their use, complementarity, and interoperability in a way that is as impartial and objective as possible. Such advice serves a practical purpose, as users switching between software suites during the structure determination process – a common practice in all but the most straightforward projects – may encounter issues related to differences in the underlying prior information (restraint dictionaries). Anticipating such issues can help mitigate problems, even if the solution is as simple as ensuring to perform proper local validation as opposed to judging “success” purely based on global refinement statistics.

Manual evaluation of individual restraints is not something that we would generally expect a typical user to routinely perform. Rather, we find awareness of validation tools such as those provided in Privateer and Coot to be more valuable.

### Software Development

In the course of this study, we sometimes found differences between restraint generators that exceeded the differences that might be expected due to the varying designs of the programs (see supplementary information). This was particularly apparent in the generated conformer. Rather than taking these differences for granted and reporting on them in this work as-is, we reached out to the developers of AceDRG, eLBOW, Grade, and Pyrogen to discuss our findings. Many of the unexpected differences were the result of minor bugs in the software that the developers quickly remedied, thus resulting in new, improved versions of the restraint generators.

All the software tools we discussed are in active development, which means they continuously evolve and indeed improve. This evolution can be accelerated by user feedback, as exemplified here. We encourage scientific software users to reach out to developers with feedback, bug reports, and possibly feature requests. It adds value to software that benefits the entire user community.

### Design Guidelines: A Proposal

While a generic dictionary output will simply work in many cases, there are enough particularities with pyranose monosaccharides to make a case for introducing a more tailored design without breaking compatibility.

In order to make model fitting easier and start refinement from a stable conformation, the initial coordinates should always contain the overwhelmingly most probable ring conformation, which for pyranoses – as saturated rings – will be a chair. All the software used in this work have been shown to be capable of doing this. Conformers should be checked with *Privateer* [15] if possible. Any anomalies should be reported to the software author.

In terms of geometric restraints, there seems to be moderate consistency (high consistency if only the non-hydrogen atoms are considered) among the programs using small molecule databases as their source of chemical knowledge. Here we were able to confirm previously reported notable differences between those and programs using other paradigms or older data based on examination of molecular databases [11, 12]. We recommend that AceDRG, Grade, Pyrogen, or eLBOW running in Mogul mode are used as long as they are available to the user, as the consistency between the restraints they generate and what the wwPDB are using for validation will keep outliers to a minimum when working with low-resolution data.

Torsion restraints are one aspect where the programs clearly differ, with results showing anything between none and a complete set of restraints. As these are useful tools at low resolution, we recommend that torsion restraints are checked and, if unsatisfactory, patched with *Privateer* [15], which will add a set of unimodal ring torsion restraints based on the starting coordinates.

Due to the extendable nature of the restraint dictionaries, we recommend that additional carbohydratespecific annotations are added to the restraint dictionaries – *e.g.,* synonyms such as IUPAC [51] names, WURCS [52, 53], or Carbohydrate Building Block (CBB) code (IUPAC glycoinformatics task group, Martin Frank, personal communication). This sort of added information could prove useful in further bridging between glycoinformatics and structural glycobiology [54]. We believe this is an area where transdisciplinary action has the potential to benefit both communities.

## Supplementary Material

Supp Info
