# Supplementary material for: Towards Consistency in Geometry Restraints for Carbohydrates in the Pyranose form: Modern Dictionary Generators Reviewed
Source: Curr Med Chem. Author manuscript; Available in PMC 2022 Mar 17. (PMC7612510; doi:10.2174/0929867328666210902140754)
Supplement: Supp Info [file EMS143790-supplement-Supp_Info.pdf]

Supplementary Material

Towards Consistency in Geometry Restraints for Carbohydrates in the Pyranose form: Modern Dictionary Generators Reviewed

Robbie P. Joosten[1], Robert A. Nicholls[2] and Jon Agirre[3,*]

[1]Oncode Institute and Division of Biochemistry, Netherlands Cancer Institute, Plesmanlaan 121, 1066 CX Amsterdam, The Netherlands; [2]Structural Studies, MRC Laboratory of Molecular Biology, Francis Crick Avenue, Cambridge CB2 0QH, England; [3]York Structural Biology Laboratory, Department of Chemistry, University of York, YO10 5DD, England

Supplementary Table 1. Summary of the validation data (anomalies in bold) calculated by Privateer on the conformers that were produced at the start of the present work, before any modifications were made to the restraint generators. All validation criteria were satisfied after the modifications made by developers. The individual programs were asked to produce an output PDB file containing the starting coordinates, and this was supplied to Privateer [1]. Do the atom names follow the naming convention in the input chemical component file [2]? Does the ring's conformation in the starting coordinates match the minimal energy conformation? Cases where the programs disagree have been marked in bold [3]. Does the monosaccharide's stereochemistry match the one specified in the input chemical component file? If it does not, what cases have registered issues.

	Acedrg	eLBOW (all methods)	Grade	Pyrogen
Error/crash	No	No	No	No
Atom names[1]	OK	All OK	OK	OK
Conformation[2]	BGC: $^4C_1$<br>**GAL: $^5S_1$**<br>**2FG: $^3S_1$**<br>GCN: $^4C_1$<br>**NAG: $^3S_1$**<br>**SIA: $^OS_2$**	BGC: $^4C_1$<br>GAL: $^4C_1$<br>2FG: $^4C_1$<br>GCN: $^4C_1$<br>NAG: $^4C_1$<br>SIA: $^1C_4$	BGC: $^4C_1$<br>GAL: $^4C_1$<br>2FG: $^4C_1$<br>GCN: $^4C_1$<br>NAG: $^4C_1$<br>SIA: $^1C_4$	**BGC: $^1C_4$**<br>**GAL: $^OS_2$**<br>2FG: $^4C_1$<br>**GCN: $B^{3,O}$**<br>**NAG: $^1S_5$**<br>**SIA: ^2S_O**
Stereochemistry[3]	OK	All OK	OK	**GCN: β**<br>**SIA: β**

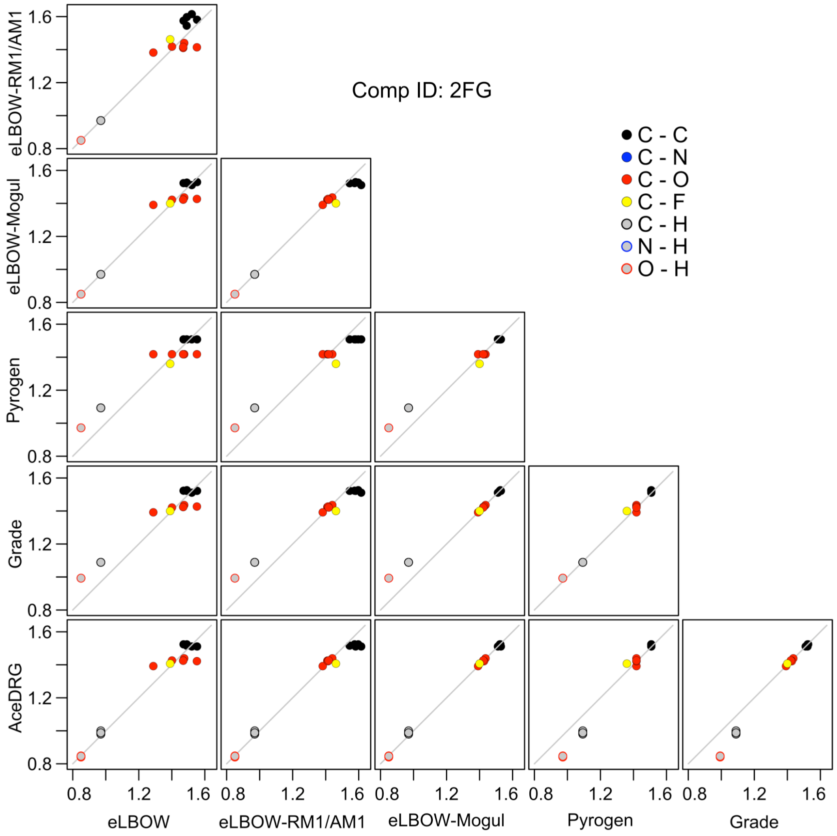

Supplementary Figure 1: *Versus* plots for equivalent bond lengths in 2FG across the different programs. Distances are specified in Ångströms.

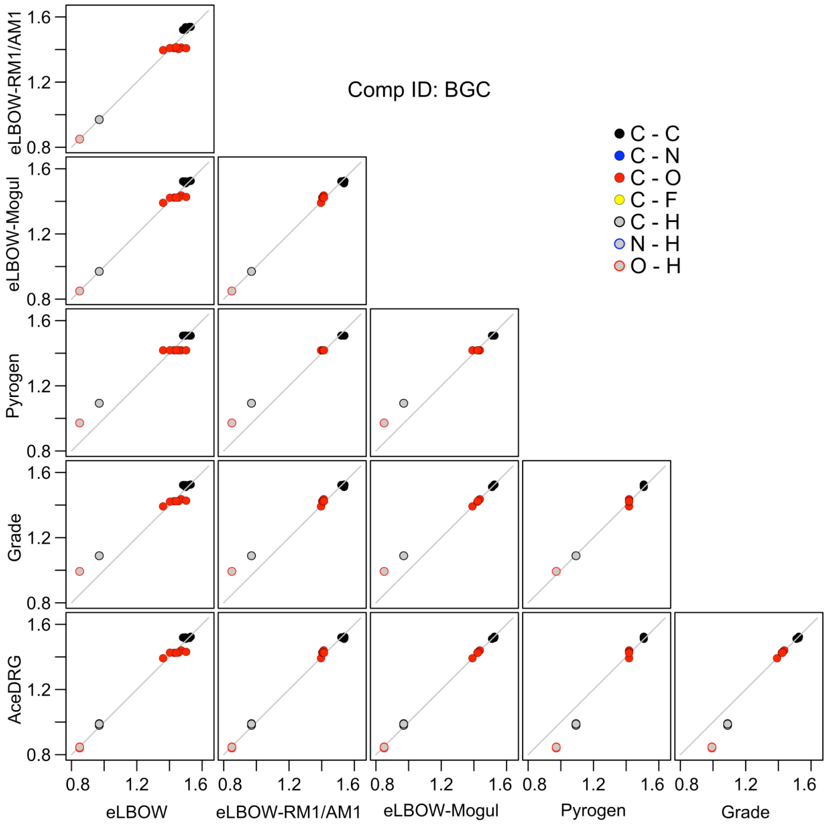

Supplementary Figure 2. *Versus* plots for equivalent bond lengths in BGC across the different programs. Distances are specified in Ångströms.

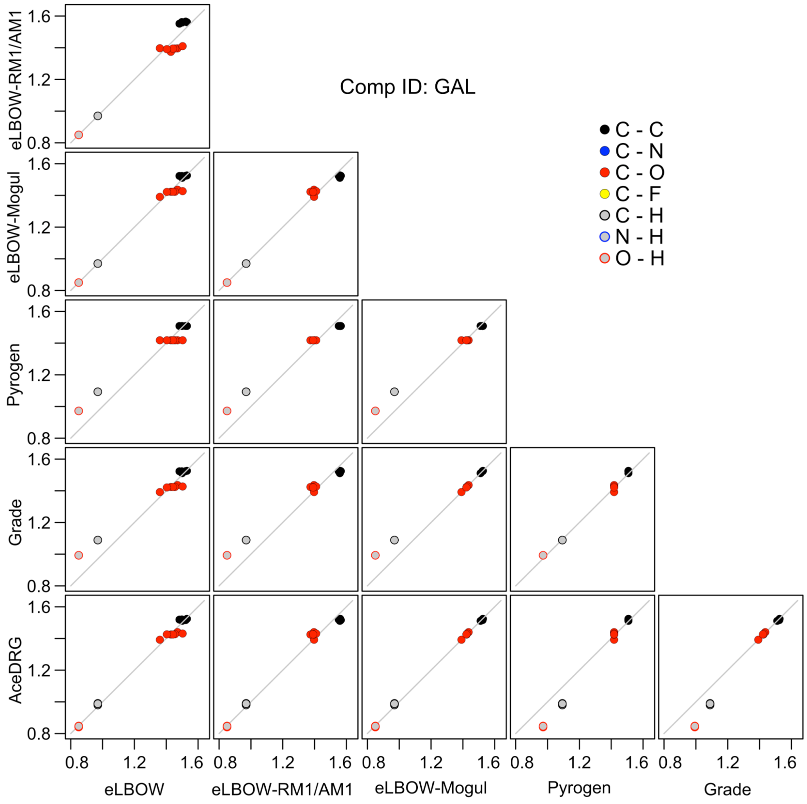

Supplementary Figure 3. *Versus* plots for equivalent bond lengths in GAL across the different programs. Distances are specified in Ångströms.

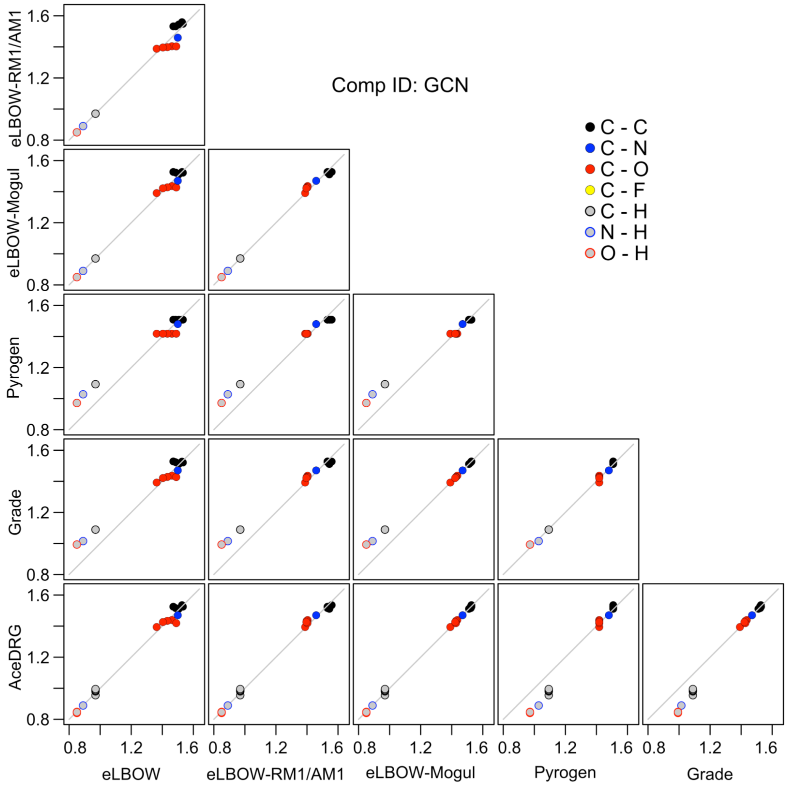

Supplementary Figure 4. *Versus* plots for equivalent bond lengths in GCN across the different programs. Distances are specified in Ångströms.

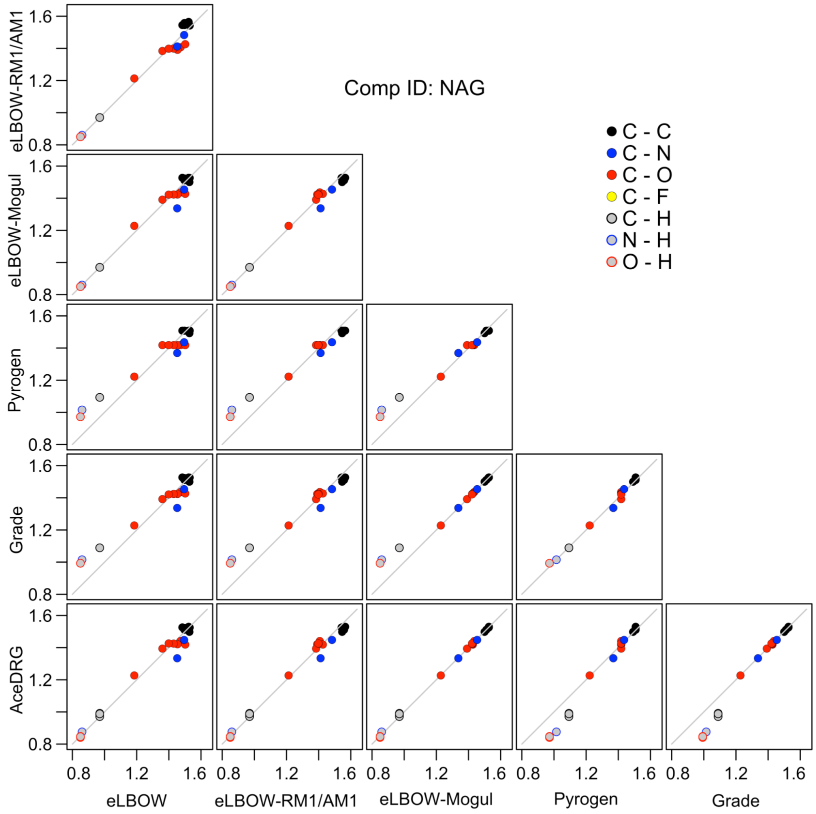

Supplementary Figure 5. *Versus* plots for equivalent bond lengths in NAG across the different programs. Distances are specified in Ångströms.

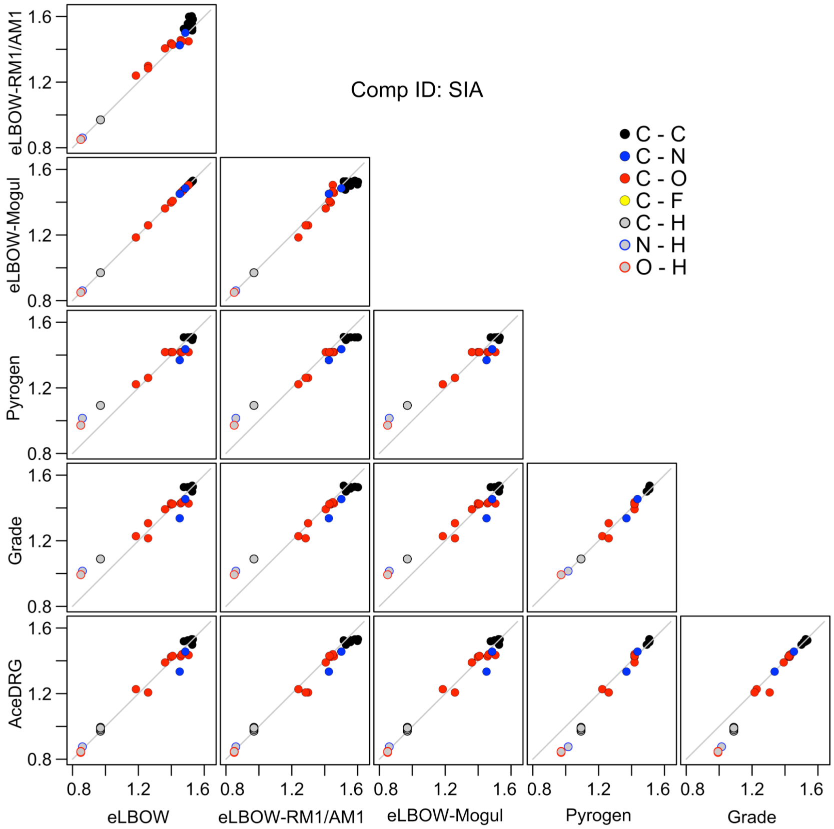

Supplementary Figure 6. *Versus* plots for equivalent bond lengths in SIA across the different programs. Distances are specified in Ångströms.

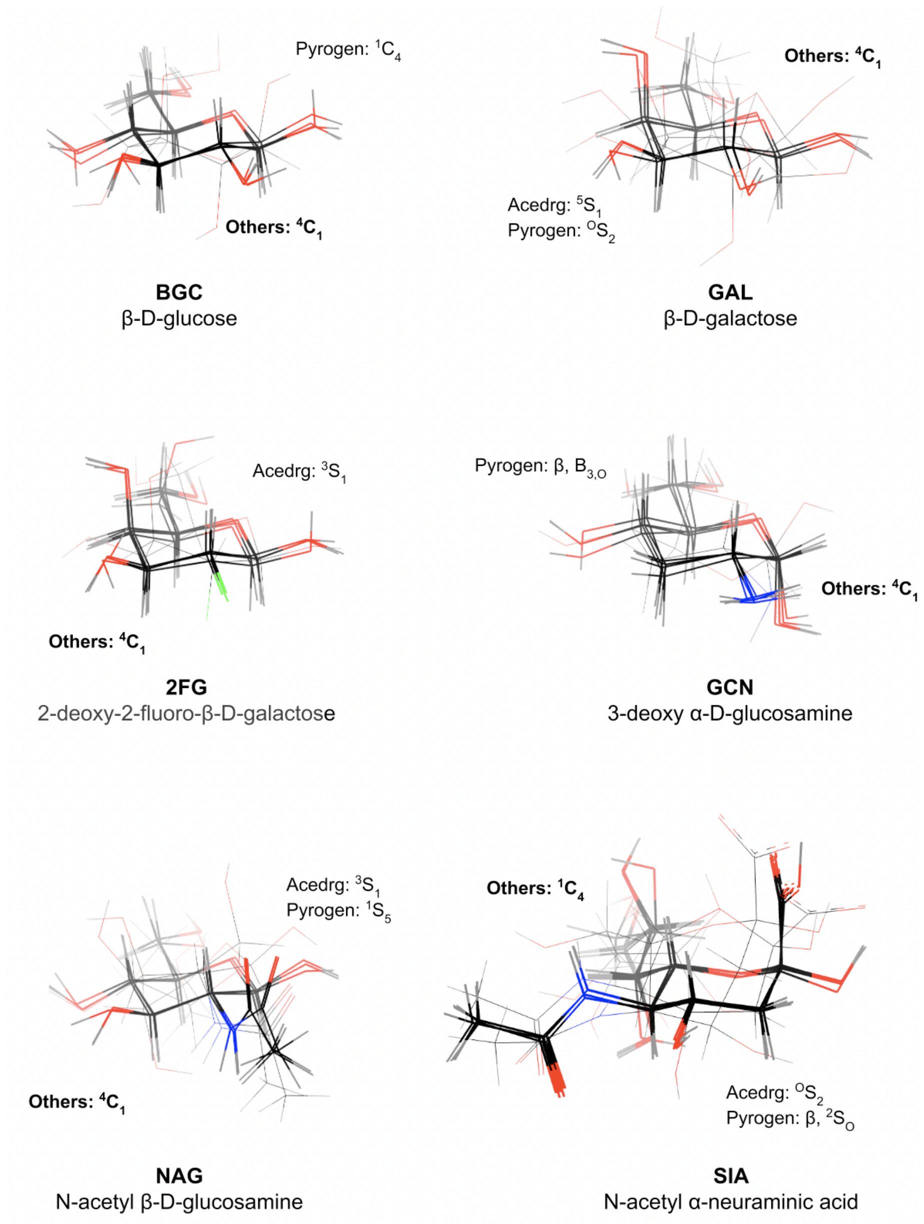

Supplementary Figure 7. Superposition of the starting coordinates produced by all programs before any modifications were made. Expected minimal-energy ring conformations are shown as thick lines, and high-energy conformers as thin lines. The expected minimal-energy ring conformations, shown as thick lines, represent the most probable conformations, thus making suitable starting coordinates for most cases. High-energy conformations on the other hand represent a highly improbable state of the molecule. Figure and superposition produced with CCP4mg [46].
